# Temozolomide toxicity operates in a xCT/SLC7a11 dependent manner and is fostered by ferroptosis

**DOI:** 10.18632/oncotarget.11858

**Published:** 2016-09-06

**Authors:** Tina Sehm, Manfred Rauh, Kurt Wiendieck, Michael Buchfelder, IIker Y. Eyüpoglu, Nicolai E. Savaskan

**Affiliations:** ^1^ Translational Cell Biology & Neurooncology Lab, Department of Neurosurgery, Universitätsklinikum Erlangen, Medical School of The Friedrich Alexander University of Erlangen-Nürnberg (FAU), Erlangen, Germany; ^2^ Department of Pediatrics and Adolescent Medicine, Universitätsklinikum Erlangen, Medical School of The Friedrich Alexander University of Erlangen-Nürnberg (FAU), Erlangen, Germany; ^3^ Department of Spinal Colum Therapies, Kliniken Dr. Erler, Nürnberg, Germany; ^4^ BiMECON Ent. Berlin, Germany

**Keywords:** brain tumor, cell death, ferroptosis, glioma niche, apoptosis

## Abstract

The glutamate exchanger xCT (SLC7a11) is causally linked with the malignancy grade of brain tumors and represents a key player in glutamate, cystine and glutathione metabolism. Although blocking xCT is not cytotoxic for brain tumors, xCT inhibition disrupts the neurodegenerative and microenvironment-toxifying activity of gliomas. Here, we report on the use of various xCT inhibitors as single modal drugs and in combination with the autophagy-inducing standard chemotherapeutic agent temozolomide (Temodal/Temcad®, TMZ). xCT overexpressing cells (xCT^OE^) are more resistant to the FDA and EMA approved drug sulfasalazine (Azulfidine/Salazopyrin/Sulazine®, SAS) and RNAi-mediated xCT knock down (xCT^KD^) in gliomas increases the susceptibility towards SAS in rodent gliomas. In human gliomas, challenged xCT expression had no impact on SAS-induced cytotoxicity. Noteworthy, other xCT inhibitors such as erastin and sorafenib showed enhanced efficacy on xCT^KD^ gliomas. In contrast, cytotoxic action of TMZ operates independently from xCT expression levels on rodent gliomas. Human glioma cells with silenced xCT expression display higher vulnerability towards TMZ alone as well as towards combined TMZ and SAS. Hence, we tested the partial xCT blockers and ferroptosis inducing agents erastin and sorafenib (Nexavar®, FDA and EMA-approved drug for lung cancer). Noteworthy, xCT^OE^ gliomas withstand erastin and sorafenib-induced cell death in a concentration-dependent manner, whereas siRNA-mediated xCT knock down increased susceptibility towards erastin and sorafenib. TMZ efficacy can be potentiated when combined with erastin, however not by sorafenib. Moreover, gliomas with high xCT expression are more vulnerable towards combinatorial treatment with erastin-temozolomide. These results disclose that ferroptosis inducers are valid compounds for potentiating the frontline therapeutic agent temozolomide in a multitoxic approach.

## INTRODUCTION

Malignant gliomas are the most lethal primary brain tumor in children and adults. The median survival time from diagnosis on is approximately 14 months [1, 2]. From those, glioblastomas (GBM; WHO grade IV) are hallmarked by features such as uncontrolled cellular proliferation, diffuse infiltration, resistance to apoptosis, angiogenesis and rampant genomic instability [1]. The current standard of care for newly diagnosed GBM in patients includes surgery as a first-line therapy, followed by radiotherapy and adjuvant temozolomide (TMZ) treatment. This regiment confers still a median survival time of only 14.6 months compared with 12.2 months for patients receiving radiotherapy alone [1]. Although temozolomide (TMZ, brand names Temodal® or Temcad®) offers some hope to GBM patients with increasing progression free and overall survival of a few months, a best 5-year survival rate of only 9.8% is currently achieved.

TMZ, a readily 194 Da lipophilic molecule is an orally available DNA alkylating agent of the imidazotetrazine class. Converted to methyltriazen-1-yl imidazole-4-carboxamide (MTIC), TMZ acts cytotoxic via mispair and futile mismatch repair loop leading to apoptotic and autophagic cell death [3]. Moreover, cells respond towards TMZ treatment with increased xCT expression as a sign of endoplasmatic cell stress [4]. Evasion of cell death and development of redox stability is one of the hallmarks of cancers and promotes tumorigenesis as well as chemo-resistance. Apoptosis, the programmed form of cell death can be engaged via the intrinsic (mitochondrial) or extrinsic (death receptor) pathway. Hence, recent studies indicated a distinct non-apoptotic cell death mechanism which can be rescued by iron chelation or blockage of iron uptake [5]. This cell death mechanism is termed ferroptosis and has unique morphological, biochemical and functional features [6]. Recently, it has been shown that the glutamate-cystine exchanger xCT (SLC7a11) appears to be essential in the process of ferroptosis in some cell types and novel small molecules such as erastin and sorafenib have been identified as xCT inhibitors [5, 7]. Hence, since xCT plays a relevant role in tumor-microenvironment interactions, i.e. inducing of peritumoral neuronal cell death and perifocal edema [8, 9], there is a quest for compounds inhibiting this transporter as novel anti-cancer agents [7]. Blocking xCT transporter could therefore lead to both, ferroptosis and reduction of the clinically dreaded perifocal edema. However, it is still unclear whether the xCT signaling pathway interacts with TMZ treatment, independent from its DNA alkylating potency.

Here, we tested the role of xCT in temozolomide-induced cell death. We found that TMZ efficacy depends on the xCT expression in human gliomas. Further, the gliomatoxic impact of TMZ can be potentiated by ferroptosis inducing agents such as erastin and sorafenib.

## RESULTS

### xCT overexpressing gliomas are resistant to sulfasalazine (SAS)

**Figure 1:**
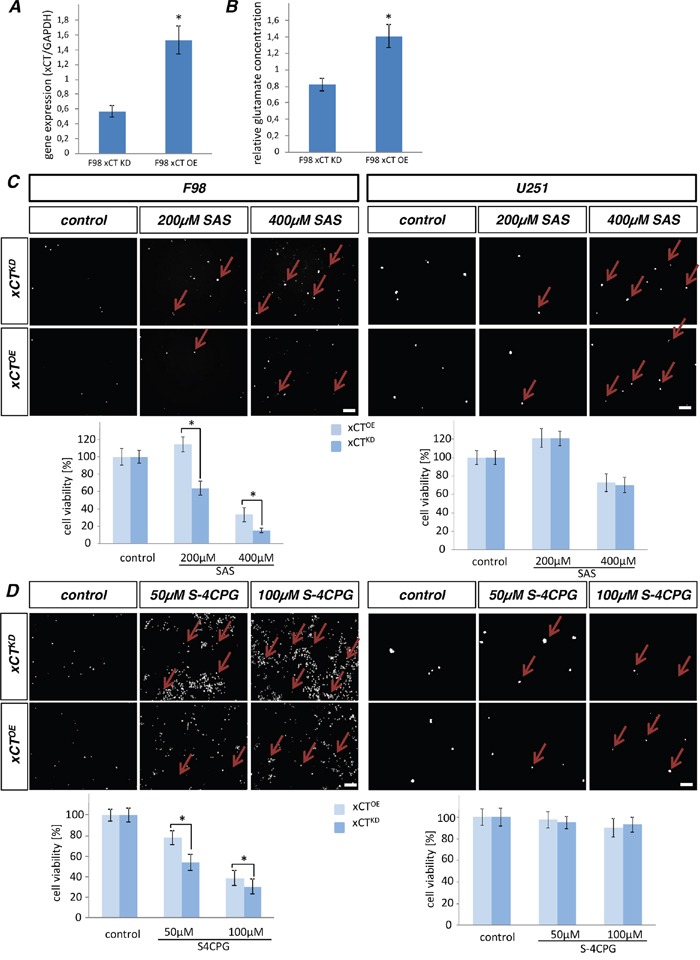
Sulfasalazine acts on human gliomas xCT-dependent **A.** Examination of the xCT levels in F98 cells. F98 xCT^KD^ cells express lower xCT transcripts than F98 xCT^OE^ cells. Differences were considered statistically significant with values mean ± SEM (n ≥ 8 per group; unpaired two-sided *t*-test, p < 0.05). **B.** Extracellular glutamate release in F98 modulated cells were measured and revealed that F98 xCT^KD^ cells secrete reduced glutamate levels than F98 xCT^OE^ cells. Differences were considered statistically significant with values mean ± SEM (n ≥ 3 per group; unpaired two-sided *t*-test, p < 0.05). **C.** Cell death of glioma cells with overexpressing xCT (xCT^OE^) and RNAi mediated silenced xCT (xCT^KD^) were examined after SAS treatment with propidium iodide (PI). The investigated species were rat (F98) and human (U251) glioma cells. Dead cells take up the PI dye and appear as white stained nuclei (arrows). Cell viability was significantly reduced after increasing SAS applications in F98 cells. xCT^OE^ cells display higher resistance to SAS treatment compared to xCT^KD^ cells. Human U251 cells with xCT overexpression or xCT silencing showed no differences in cell viability and cell death following SAS treatment. Scale bar represents 100 μm. Differences were considered statistically significant with values mean ± SEM (n ≥ 4 per group; two-way anova, p < 0.05). **D.** Analysis of cell death on rat and human glioma xCT^OE^ and xCT^KD^ following S-4-CPG treatment. Cell death of glioma cells with overexpressing xCT (xCT^OE^) and RNAi mediated silenced xCT (xCT^KD^) were examined after S-4-CPG treatment with propidium iodide (PI). Dead cells appear as white dots (arrows). Cell viability was significantly reduced after increasing SAS applications in F98 cells. xCT^OE^ cells display higher resistance to S-4-CPG treatment compared to xCT^KD^ cells. Human U251 cells with xCT overexpression or xCT silencing showed no differences in cell viability and cell death following SAS treatment. Scale bar represents 100 μm. Differences were considered statistically significant with values mean ± SEM (n ≥ 4 per group; two-way anova, p < 0.05).

First, we investigated the expression levels of xCT in xCT-modulated glioma cells. Thus, F98 RNAi mediated xCT silenced gliomas (xCT^KD^) express less xCT in comparison to xCT overexpressing cells (xCT^OE^) (Figure 1A). To investigate the consequences of deranged xCT expression we measured the extracellular glutamate release. We found that F98 xCT^KD^ cells secrete significantly less glutamate compared to F98 xCT^OE^ cells (Figure 1B). Further, we examined the effects of the reported xCT inhibitors SAS and S-4-CPG, although not exclusively specific, on xCT-modulated brain-derived cancer cells. Human (U251) and rat glioma cells (F98) overexpressing xCT (xCT^OE^) or RNAi mediated xCT silenced gliomas (xCT^KD^) were examined for cell death and cell viability after SAS treatment. Following high concentration of SAS we monitored increased cell death and reduced cell viability in F98 xCT^KD^ cells (Figure 1C). At 200 μM SAS decreased cell viability already to over 40%. The IC_90_ was reached at 400 μM SAS. F98 xCT^OE^ cells appeared to be more resistant towards SAS compared to xCT^KD^, and survival of xCT^OE^ cells was only reduced at 400 μM SAS in comparison to the untreated controls (Figure 1C). We investigated also cell death response and viability of the human glioma cells (U251) following SAS treatment. Solely high concentration of SAS led to a significant reduction of the cell viability about 40%. Remarkable is that there was no differences in cell viability and cell death between xCT^KD^ and xCT^OE^. Further we analysed cell death and cell viability after application of the xCT inhibitor S-4-CPG (Figure 1D). A growth inhibitory effect of S-4-CPG on F98 cells were observed first at 50 μM. Noteworthy, F98 xCT^OE^ cells exhibited higher resistance towards S-4-CPG compared to xCT^KD^ cells (20% cell viability rate vs. 45%) (Figure 1D). Furthermore we examined the effects of S-4-CPG on U251 cells. Noteworthy, there was no significant cell viability reduction at high S-4-CPG concentrations independently of the xCT expression levels (between xCT^KD^ and xCT^OE^).

**Figure 2:**
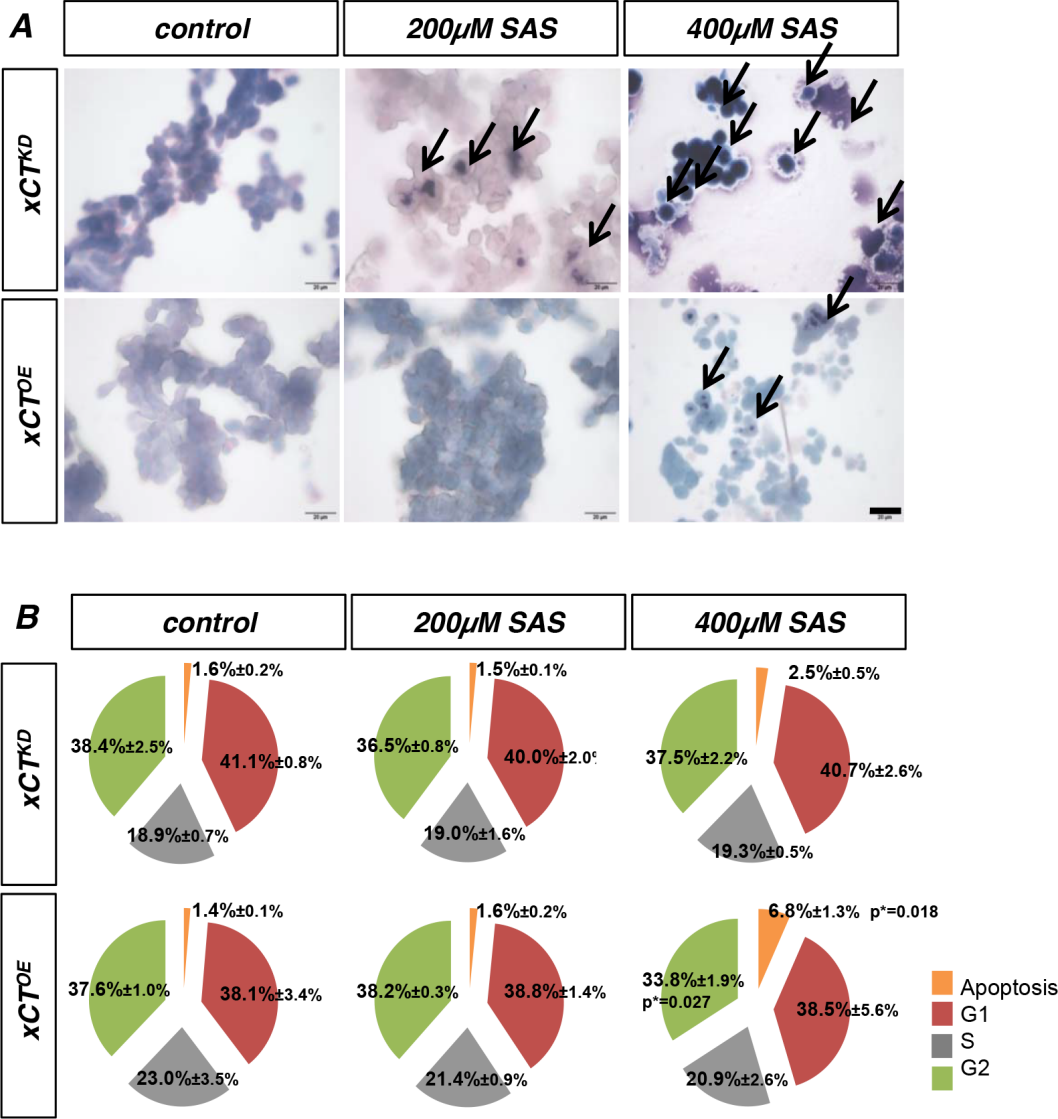
The impact of Sulfasalazine on xCT expressing and xCT silenced gliomas **A.** Wright staining reveals apoptotic cell death in F98 xCT^OE^ and xCT^KD^ cells treated with SAS. The staining of dead cells (black arrows) confirms cell death data found with PI staining and cell viability measurements. Scale bar represents 100 μm. **B.** F98 xCT^OE^ and xCT^KD^ glioma cells were treated with SAS and the cell cycle was investigated. Apoptosis (Sub-G1; apoptotic bodies) increased in a concentration dependent manner. Values are given as mean ± SD (n ≥ 3 per group; unpaired two-sided *t*-test, p < 0.05).

Hence, the histological Wright stain displayed the morphological features of apoptotic cells and confirmed the cell death and cell viability measurements (Figure 2A). Furthermore, we applied SAS to xCT^OE^ and xCT^KD^ cells again and conducted a cell cycle analysis with 7-AAD (Figure 2B; Supplementary Figure S1A). 400 μM SAS significantly increased apoptotic cell death in comparison to untreated controls. Other cell cycle parameters were not significantly altered following SAS treatment (Figure 2B).

### The impact of temozolomide is dependent on xCT in human gliomas

**Figure 3:**
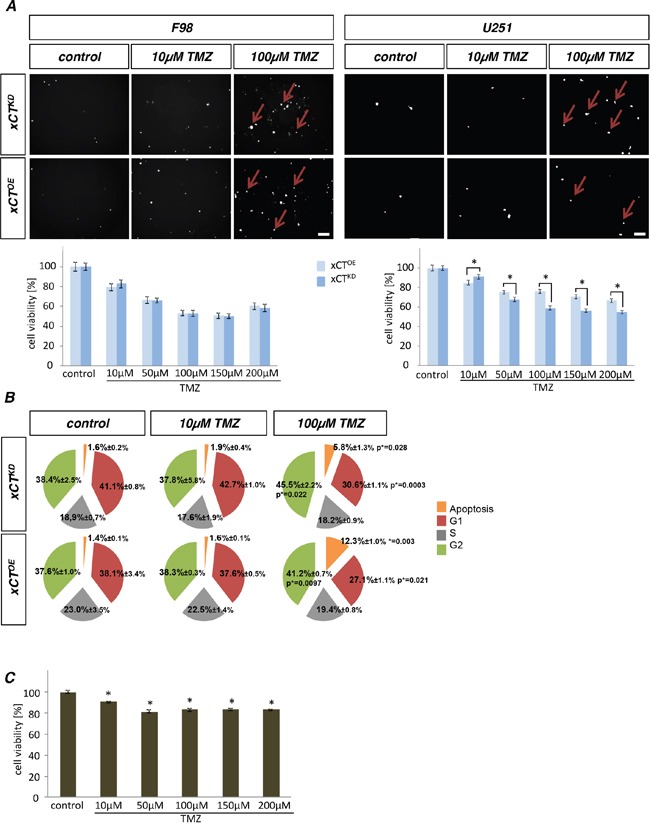
Temozolomide acts on human glioma cells in a xCT-dependent manner **A.** F98 and U251 xCT^OE^ and xCT^KD^ glioma cells were treated with various concentrations of temozolomide (TMZ) followed by cell death monitoring with propidium iodide (PI, red arrows). Scale bar represents 100 μm. Differences were considered statistically significant with values mean ± SEM (n ≥ 4 per group; two-way anova, p < 0.05). **B.** A cell cycle analysis was performed on xCT^OE^ and xCT^KD^ cells treated with TMZ. Apoptosis increased in a concentration dependent manner. Values are given as mean ± SD (n ≥ 3 per group; unpaired two-sided *t*-test, p < 0.05). **C.** Various concentrations of TMZ were applied to primary rodent astrocytes and cell survival was determined. Values are given as mean ± SEM (n ≥ 4 per group; unpaired two-sided *t*-test, p < 0.05).

We continued to analyse the influence of the standard chemotherapeutic agent TMZ on xCT-modulated glioma cells. Cell death on glioma cells as well as cell viability was monitored after TMZ treatment (Figure 3A). Concentrations of 10 μM TMZ were already toxic on both xCT^OE^ and xCT^KD^ cells in rat and human tested glioma cell lines. The toxic effects on glioma cells increased with elevated concentrations of TMZ up to 100μM. The IC_50_ was reached at 150 μM to 200 μM TMZ. Noteworthy, for F98 cells TMZ was impacting cell viability of xCT^OE^ and xCT^KD^ cells in the same manner (Figure 3A). U251 xCT^KD^ were more susceptible to 100 μM TMZ than xCT^OE^ cells. We further investigated the cell cycle of F98 xCT^OE^ and xCT^KD^ cells after TMZ treatment (Figure 3B; Supplementary Figure S1B). TMZ increased apoptotic cell death in a concentration-dependent manner. We observed significant reduction of the G1 phase and prolonged G2-phases following TMZ, whereas the S-phases of xCT^OE^ and xCT^KD^ cells did not change significantly after TMZ treatment.

To test whether TMZ is generally toxic to non-transformed differentiated brain cells, we investigated the toxicity profile of various TMZ concentrations on primary astrocytes (Figure 3C). Within a wide range of various TMZ levels primary astrocytes displayed only minor toxic effects (Figure 3C). Solely highest TMZ concentrations reduced cell growth of primary astrocytes to an extent of 20% compared to controls (Figure 3C).

### SAS potentiates chemo-sensitivity of temozolomide in xCT knockdown gliomas

**Figure 4:**
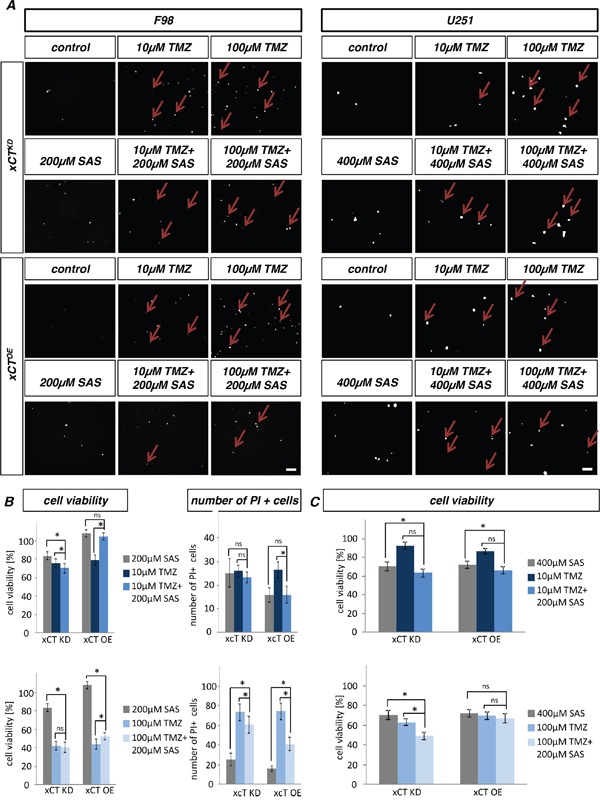
Temozolomide treatment in combination with sulfasalazine **A.** Cell death of RNAi mediated xCT knock down cells (xCT^KD^) and overexpressing xCT (xCT^OE^) cells was analyzed with propidium iodide (PI) after sulfasalazine (SAS) and temozolomide (TMZ) treatment. Scale bar represents 100 μm. **B.** Cell viability determined by MTT assay monitored in F98 cells. In F98 xCT^KD^ cells 10 μM TMZ plus 200 μM SAS showed an additive cell death effect. Differences were considered statistically significant with values as mean ± SEM (n ≥ 4 per group; one-way anova, p < 0.05). PI positive cell counting revealed no additive cell death effect. Differences were considered statistically significant with values given as mean ± SEM (n ≥ 15 per group; n = 15; unpaired two-sided *t*-test, p < 0.05). **C.** In U251 xCT^KD^ cells 100 μM TMZ plus 400 μM SAS showed potentiated cell death effects. Differences were considered statistically significant with values given as mean ± SEM (n ≥ 4 per group; one-way anova, p < 0.05).

Hence, we studied the multimodal treatment with the xCT inhibitor SAS and the standard therapeutic agent TMZ in rat (F98) and human (U251) glioma cell lines. Combined SAS and TMZ treatment increased cell death in F98 and U251 xCT^KD^ cells and reduced cell survival (Figure 4A, 4B, 4C). F98 xCT^KD^ cells showed higher vulnerability compared to F98 xCT^OE^ cells when treated solely with SAS (Figure 4B). U251 xCT^KD^ and xCT^OE^ cells responded to the same extent to SAS treatment (Figure 4C).

Following single TMZ treatment both F98 xCT^OE^ and F98 xCT^KD^ cells displayed similar cell viability rates without any differences in comparison to U251, here changes were visible (Figure 4B, 4C). The multimodal treatment approach with 10 μM TMZ and 200 μM SAS showed a significant additive effect in terms of cell death by F98 xCT^KD^ (Figure 4B). The combination of SAS and TMZ on F98 xCT^KD^ appeared more toxic than single SAS or TMZ application. The staining for PI showed necrotic cells. In particular multimodal treatment schemes were not more effective in killing rat glioma cells in comparison to single compound applications. In contrast, the multimodal treatment approach displayed significantly additive effect at 100 μM TMZ with 400 μM SAS in the xCT^KD^ in case of human glioma cells.

**Figure 5:**
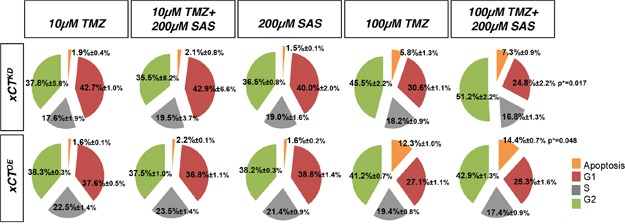
Cell cycle analysis for temozolomide treatment in combination with Sulfasalazine **A.** Cell cycle analysis of F98 and U251 cells treated with ± temozolomide (TMZ), ± sulfasalazine (SAS) and combinations of TMZ and SAS. Values are given as mean ± SD (n ≥ 3 per group; unpaired two-sided *t*-test, p < 0.05).

Next, we performed cell cycle analysis after TMZ and SAS treatment regimens (Figure 5C, Supplementary Figure S1C). Noteworthy, there were no alterations in cell cycle parameters after single TMZ and SAS as well as after combined SAS and TMZ treatment approaches (Figure 5C). However, in xCT^KD^ cells we found increased apoptosis after 200 μM SAS and 100 μM TMZ application (Figure 5C).

### Efficacy of erastin and sorafenib is dependent on xCT in gliomas

**Figure 6:**
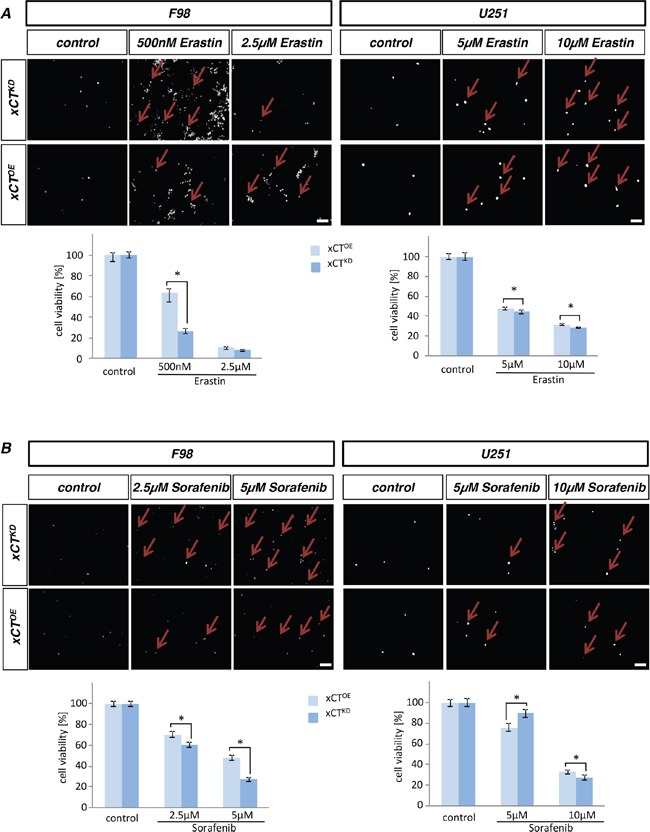
Erastin and sorafenib induced cell death depends on xCT levels **A.** Cell death of rodent (F98) and human (U251) xCT^OE^ and xCT^KD^ cells were examined after adding erastin. Propidium iodide (PI) was facilitated for dead cell detection (red arrows). **B.** Sorafenib application on xCT overexpressing (xCT^OE^) cells and RNAi mediated xCT knock down cells (xCT^KD^) for rat and human glioma cells. Cell viability was measured for erastin and sorafenib on F98 and U251 cells. xCT^KD^ cells are more sensitive towards erastin and sorafenib in comparison to xCT^OE^ cells. F98 cells are more susceptible for erastin and sorafenib in comparison to human U251 cells. Scale bar represents 100 μm. Differences were considered statistically significant with values mean ± SEM (n ≥ 4 per group; two-way anova, p < 0.05).

Since the combination of SAS together with TMZ was unexpectedly less effective on glioma cells we tested two novel small molecule compounds, reported also as xCT inhibitors, in these assays. Following erastin application we monitored cell death and cell viability of human and rodent xCT^KD^ and xCT^OE^ gliomas (Figure 6A). Erastin was already toxic to F98 cells at a concentration of 500 nM. U251 revealed a tenfold higher resistance for erastin.

Interestingly, xCT^OE^ cells were more resistant towards erastin compared to xCT^KD^ cells (cell viability rates of 35% versus 70%) (Figure 6A). We next tested the influence of challenged xCT levels on sorafenib impact (Figure 6B). Sorafenib reduced significantly cell proliferation of F98 glioma cells already at 2.5 μM. U251 appeared more resistant against sorafenib, revealing first effects at 5 μM sorafenib.

Noteworthy, xCT^OE^ cells exhibited resistance towards sorafenib compared to xCT^KD^ cells (cell viability rates of 30% versus 40%) (Figure 6B). Thus, xCT^KD^ cells displayed higher susceptibility for erastin and sorafenib-induced cell death in comparison to xCT^OE^ gliomas.

### Primary astrocytes and neurons are less vulnerable to xCT inhibitors

**Figure 7:**
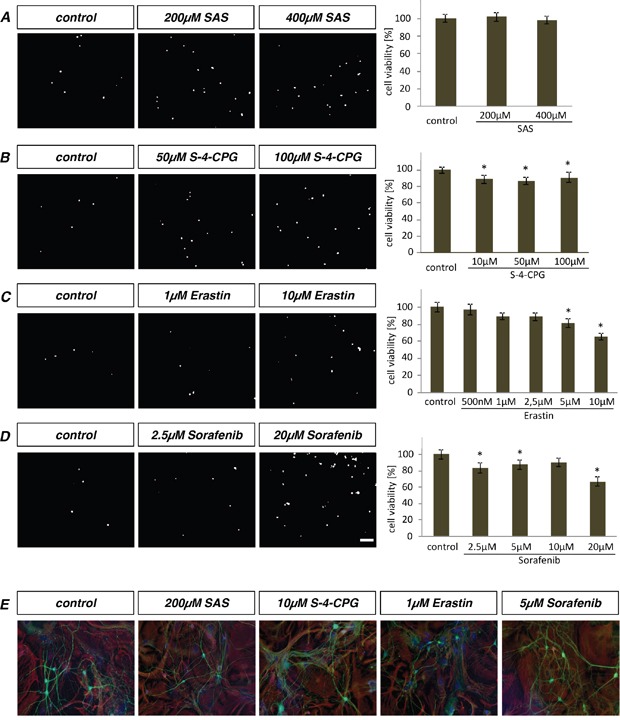
Primary astrocytes and neurons withstand toxic effects of erastin and sorafenib **A.** Primary rat astrocytes were treated with various concentrations of SAS. Cell viability was measured by propidium iodide (PI) staining. SAS has no effect on astrocytes. **B.** The impact of S-4-CPG on astrocytes. Cell viability shows minor but significant decrease. **C.** Erastin application on primary rat astrocytes displays a slightly decline in cell viability. This effect is significant at 5 μM erastin. **D.** Cell viability monitoring of sorafenib treated primary astrocytes. Scale bar represents 100 μm. Differences were considered statistically significant with values mean ± SEM (n ≥ 4 per group; unpaired two-sided *t*-test, p < 0.05). **E.** Primary neurons were treated with the xCT inhibitors, SAS, S-4-CPG, erastin and sorafenib. The mixed neurons-astrocytes culture was stained for beta-III-tubulin (revealing neurons) and GFAP (marker for astrocytes). There were no toxic effects detectable in neurons and astrocytes.

In order to investigate whether ferroptosis inducers and xCT inhibitors are generally toxic to non-transformed differentiated brain cells, we monitored the toxicity of these xCT inhibitors on primary astrocytes and neurons (Figure 7). In contrast to glioma cells, SAS application did not alter cell survival of astrocytes at the investigated concentrations (Figure 7A). In contrast, S-4-CPG displayed slightly toxic effects on astrocytes, with significant decrease in cell survival to 85% at a concentration of 100 μM (Figure 7B). Additionally, we investigated cell viability of astrocytes after erastin application (Figure 7C). Erastin-treated astrocytes showed a significant cell growth reduction at a concentration of 5 μM and cell death rate increased to 20% (Figure 7C). Finally we applied the ferroptosis-inducer sorafenib on primary astrocytes (Figure 7D). Sorafenib decreased cell viability already to 20% at a concentration of 2.5 μM (Figure 7D). Moreover, we investigated xCT inhibitors also on primary neurons. Noteworthy, SAS, S-4-CPG, erastin and sorafenib did not alter neuronal cell survival at the investigated concentrations (Figure 7E). Thus, astrocytes and neurons are less vulnerable to xCT inhibitors and ferroptosis inducers compared to glioma cells.

### Erastin induces ferroptotic cell death on xCT-modulated cells

**Figure 8:**
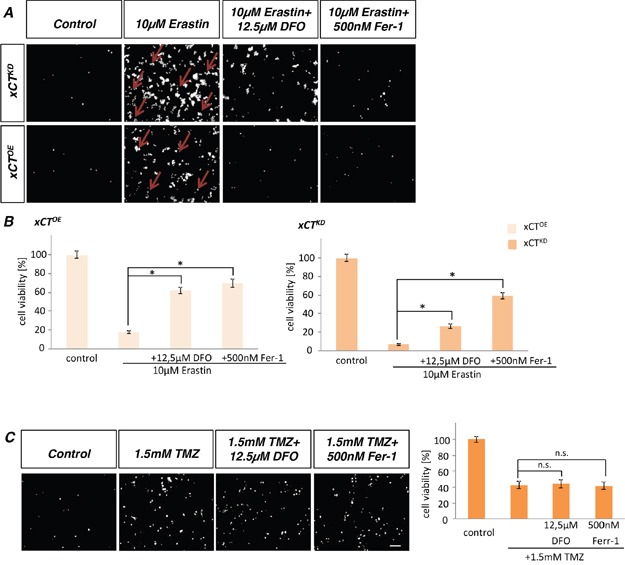
Erastin induces ferroptosis in glioma cells **A.** Cell death analysis of erastin treated glioma cells. 10 μM erastin with and without deferoxamine (DFO) or ferrostatin-1 (Ferr-1) was added to xCT^OE^ and xCT^KD^ cells. Propidium iodide (PI) staining revealed dead cells (arrows). Scale bar represents 100 μm. **B.** Quantification of cell viability revealed that xCT^KD^ cells are more sensitive to erastin in comparison to xCT^OE^ cells. Erastin induces ferroptotic cell death. Values are shown as mean ± SEM (n ≥ 4 per group; unpaired two-sided *t*-test, p < 0.05). **C.** Ferroptosis rescue assay with 1.5 mM temozolomide, ± deferoxamine (DFO) or ± ferrostatin-1 (Ferr-1) was applied to F98 cells. Noteworthy, temozolomide alone does not induce ferroptotic cell death.

To examine whether the most effective xCT inhibitor induces ferroptosis [5] on gliomas we treated rodent glioma cells with 10 μM erastin. Noteworthy, xCT^KD^ cells were highly susceptible to erastin with cell death rates of 95%. Conversely, xCT^OE^ cells displayed resistance towards erastin after 24 h treatment (Figure 8A, 8B). These data were further confirmed by ferroptosis rescue experiments (Figure 8B). Deferoxamine (DFO) and ferrostatin-1 (Fer-1) are both known iron chelator and inhibitors of ferroptosis with the potential to prevent erastin-induced accumulation of cytosolic and lipid ROS. Here, we could demonstrate that DFO and Fer-1 clearly rescued erastin-induced cell death in xCT^OE^ and xCT^KD^ glioma cells. Moreover, xCT^KD^ were more sensitive to ferroptosis compared to xCT^OE^ gliomas (Figure 8B).

### Temozolomide does not induce ferroptosis

Futher, we investigated ferroptotic cell death in rodent glioma cells following TMZ treatment. For this, we performed ferroptosis rescue experiments with DFO and Fer-1. Noteworthy, neither DFO nor Fer-1 application could rescue TMZ-induced cell death (Figure 8C).

### Multitoxicity with erastin and sorafenib increase efficacy of temozolomide

**Figure 9:**
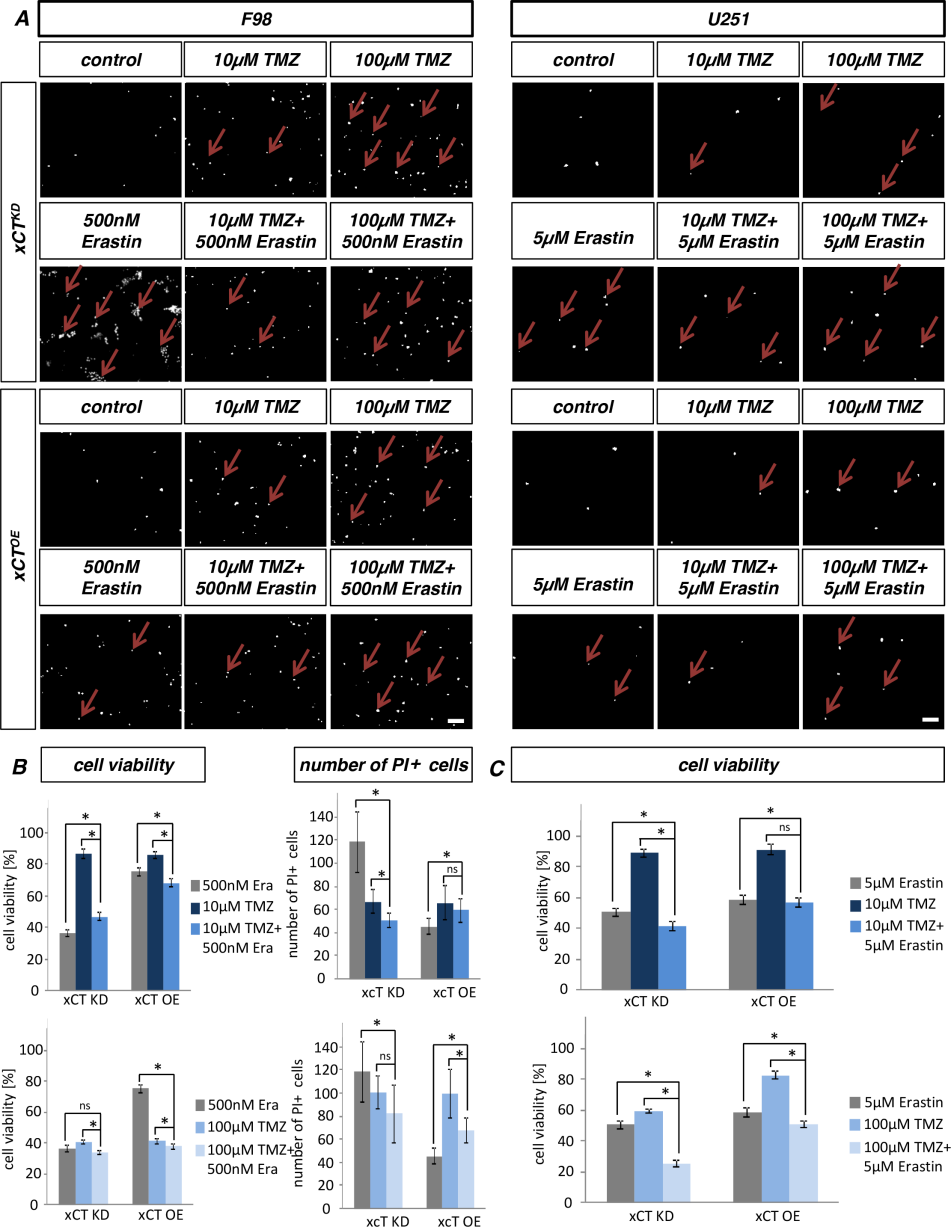
Multicytotoxic approach with temozolomide combined with erastin **A.** Cell death analysis of erastin and temozolomide multitoxic combination treatment. xCT^KD^ and xCT^OE^ cells originated from F98 and U251 cells were treated with erastin alone or in combination with TMZ. Cell death was determined with propidium iodide (PI). Scale bar represents 100 μm. **B.** Cell viability assay of erastin and temozolomide multitoxic combination treatment. Cell growth was monitored in rodent xCT^OE^ and xCT^KD^ cells treated with 500 nM erastin and TMZ. The combination of TMZ and erastin was effective on xCT^OE^ cells. Additive effects are revealed in comparison to the effect of the single substances. Values are given as mean ± SEM (n ≥ 4 per group; one-way anova, p < 0.05). Note that quantification of PI positive cells does not resemble the findings of the cell viability assays. Differences were considered statistically significant with values given as mean ± SEM (n ≥ 15 per group; n = 15; unpaired two-sided *t*-test, p < 0.05). **C.** Cell viability assay of erastin and temozolomide treated human gliomas. Cell growth was monitored in human xCT^OE^ and xCT^KD^ cells treated with 500 nM erastin and TMZ. The combination of TMZ and erastin was effective on xCT^OE^ cells. Multitoxic combination approach revealed additive effects in comparison to the effect of single substances. U251 xCT^OE^ cells and xCT^KD^ cells treated with combination of 100 μM TMZ/5 μM erastin showed an additive cell death effect. Differences were considered statistically significant with values mean ± SEM (n ≥ 4 per group; one-way anova, p < 0.05.

**Figure 10:**
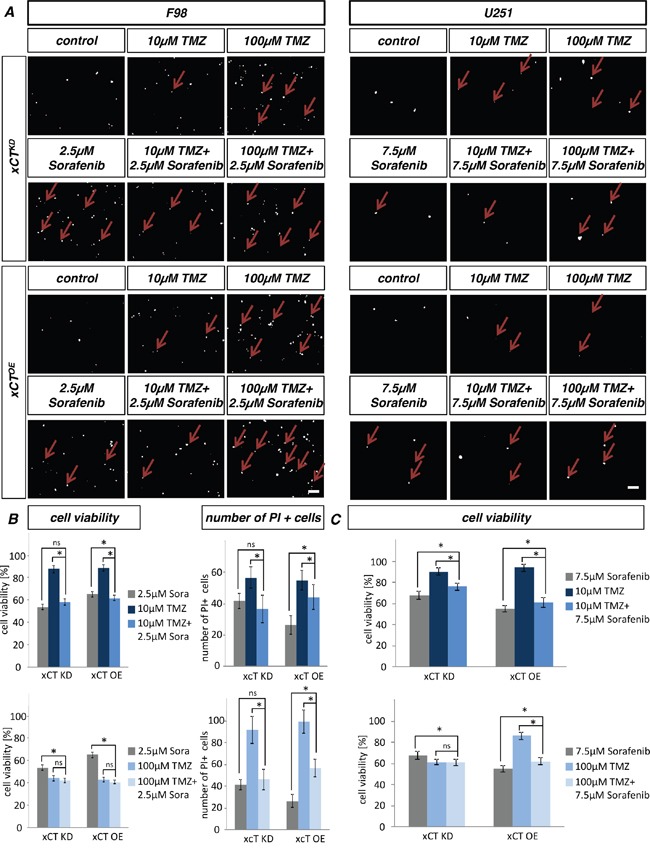
Multicytotoxic approach with Temozolomide in combination with Sorafenib **A.** Cell death of xCT^KD^ and F98 xCT^OE^ cells were determined after the treatment with sorafenib and TMZ and their combination. Propidium iodide (PI) is taken up by the dead cells thereby making dead cells visible. Scale bar represents 100 μm. **B.** Cell viability was detected in xCT^KD^ and xCT^OE^ cells treated with ±erastin and ±TMZ. Multitoxic approach with 10 μM TMZ/2.5 μM sorafenib showed additive cell death effects in xCT^OE^ cells. Values are given as mean ± SEM (n ≥ 4 per group; one-way anova, p < 0.05). Quantification of PI positive cells reveals no additive cell death effects. Differences were considered statistically significant with values shown as mean ± SEM (n ≥ 15 per group; n = 6 unpaired two-sided *t*-test, p < 0.05). **C.** Cell viability was detected in human xCT^KD^ and xCT^OE^ cells treated with ± sorafenib and ± TMZ. Multitoxic approach with 10 μM TMZ/7.5 μM sorafenib showed no additive cell death effects. Differences were considered statistically significant with values given as mean ± SEM (n ≥ 4 per group; one-way anova, p < 0.05).

Next, we investigated the multitoxic approach combining ferroptosis-inducers such as erastin and sorafenib with the alkylating agent TMZ. Rodent and human xCT overexpressing and silenced glioma cells were treated with these compounds and cell death was subsequently monitored (Figure 9, 10). TMZ treatment revealed no differences in cell viability of rodent xCT^KD^ and xCT^OE^ cells. In the case of xCT^KD^, U251 glioma cells were more sensitive against 100 μM TMZ compared to xCT^OE^ gliomas.

Single application of erastin and sorafenib alone revealed that glioma xCT^KD^ cells are more susceptible than xCT^OE^ cells. The multitoxic combinatory treatment with erastin and temozolomide (Figure 9) or sorafenib and temozolomide (Figure 10) exhibited a multiplicative cell death effect. Moreover, for F98 cells 500 nM erastin in combination 10 μM TMZ or 100 μM TMZ showed an accumulating toxic effect on xCT^OE^ cells (Figure 9A, 9B). Also, in the case of human glioma xCT^KD^ cells, this combination was effective at 5 μM erastin in combination with 10 μM TMZ. Furthermore, erastin in combination with 100 μM TMZ displayed additive toxic effects on U251 xCT^KD^ as well as xCT^OE^ cells (Figure 9C). The combination of sorafenib with TMZ revealed higher toxicity than single application of sorafenib or TMZ alone in the case of F98 xCT^OE^ for 10μM TMZ (Figure 10). Altogether, the multitoxic combinatory treatment approach with temozolomide and ferroptosis inducers revealed a multiplicative cytotoxic anti-cancer efficacy.

## DISCUSSION

Here we investigated the role of the glutamate-cystine transporter xCT in TMZ-induced cell death. Amino acid transporters in general and xCT in particular are a potentially attractive drug targets due to their pharmacological properties and reachability. In particular represents xCT a prime target for anti-cancer drugs since this transporter is crucial for glutathione homeostasis and cell survival [10–12].

**Figure 11:**
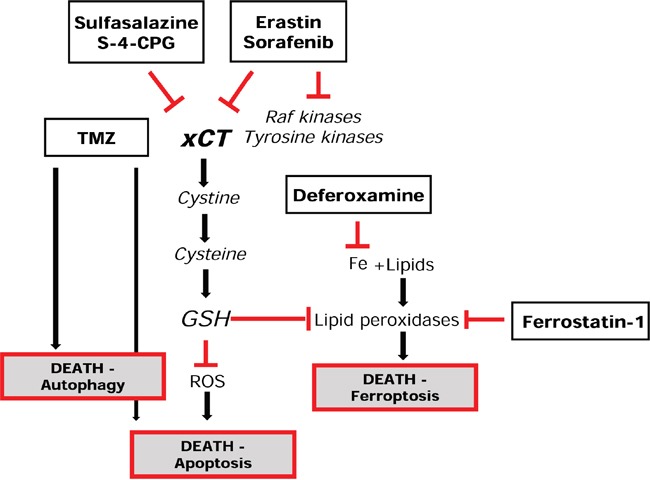
Summary of the multitox-approach with temozolomide and ferroptosis signaling Scheme of the experimental approach using various xCT inhibitors and TMZ and their contribution in cell death signaling. Note that erastin and sorafenib are no *bona fide* xCT inhibitors and have also been reported as receptor tyrosine kinase inhibitors. SAS has been clinically used for its NFkB inhibitory function for long-time. S-4-CPG is an experimental drug with certain cross affinity to metabotrophic glutamate receptors.

In this study, we demonstrate that TMZ is efficient in cell death induction and that the efficacy of TMZ depends on xCT expression levels in different glioma species. The efficacy of TMZ can be potentiated after combination with the new small molecule compounds erastin and sorafenib. We found that xCT overexpressing tumors are in particular sensitive to this multitoxic, combinatory twofold treatment strategy. The rationale for this lays in previous reports indicating that erastin and sorafenib are potent pharmacological agents inhibiting in part the glutamate antiporter xCT [7]. In fact, sorafenib (Nexavar®) is currently in use under clinical settings for the treatment of renal cell carcinoma, unresectable hepatocellular carcinoma and thyroid cancer [13, 14]. Erastin is a new small molecule compound and has been found to block xCT or indirectly inhibits signaling targets associated with xCT and other system L related transporters [7]. Both compounds are substantially more potent inhibitors than the FDA-approved drug sulfasalazine (SAS) and can in addition induce ferroptosis (Figure 11). However, both erastin and sorafenib are not specific for xCT and independent reports brought evidences that both compounds can also efficiently inhibit multiple receptor tyrosine kinases, Ras, VEGF signaling as well as Raf kinase [7]. In contrast, SAS is in clinical use primary for its NFkB inhibiting activity, although its xCT blocking actions have been evidenced in *in vitro* and *in vivo* experiments [8, 34]. For applying this multitox-approach the question remains which pharmacological function will be the main effect in humans.

Here, we hypothesized that xCT inhibition, although not fully lethal for glioma cells, can weaken the cellular resistance mechanisms against TMZ (Figure 11). The rationale for this assumption is based on the essential function of xCT in glutathione homeostasis.

xCT is central to the cellular cystine import in exchange to glutamate export which becomes reduced to cysteine and is mainly required for glutathione production [15]. Thus xCT is at the center stage for glutathione dependent redox regulation and glutamate homeostasis. Second, xCT is the main glutamate exchanger in brain cancer cells thereby creating a glutamate-rich neurotoxic microenvironment [16]. Interestingly, other glutamate transporters such as EAAT1 and EAAT2 are silenced in brain cancer and high abundant system Xc- activity result in a net balance shift towards glutamate release. Increased glutamate levels are thought to be central in advantages of glioma growth and progression. Inhibition of glutamate release via xCT inhibition profoundly decelerates the glioma phenotype *in vivo* [8, 17] and in addition mitigates tumor-induced brain swelling [8] and tumor-induced seizures [18]. It has been demonstrated in various cancer types including primary brain tumors (malignant gliomas) that xCT is a valid anti-cancer target, especially because xCT expression correlates with malignancy. First, the antiporter system xCT is abundantly expressed in glioblastoma specimens and cell lines [8, 17, 19, 20]. Second, inhibition of xCT can induce ferroptotic cell death in some cancer cells such as lymphoma cells, various epithelial carcinomas, and melanomas [21–23].

On the other side TMZ-based chemotherapy is currently standard drug in brain tumor therapy and is conceptually used as a cytotoxic agent in an uni- or multimodal therapy scheme [24]. Further, TMZ provides a survival benefit in a subset of patients with high-grade gliomas and provides the primarily palliative treatment for the vast majority of patients. However, the increase in median survival for treatment of newly diagnosed glioblastomas treated with TMZ and radiotherapy is only 2.5 months compared with radiotherapy alone [25, 26]. In addition, approximately one of five patients treated with TMZ develops clinically significant toxicity or acquired resistance, which can leave further treatment unsafe. This situation indicates that TMZ is only a modestly effective chemotherapy calling for additional strategies.

In line with this situation it would be the multicytotoxic strategy using ferroptosis inducers or xCT inhibitors for supporting already established standard chemotherapeutic agents. We tested this in conditions of temozolomide application and found some additive cytotoxic effects. An important finding is that the level of xCT in human glioma cells dictates the sensitivity and efficacy of TMZ. This indicates that TMZ actions are directly or indirectly dependent of the glutathione homeostasis and cystine/cysteine redox status (Figure 11). Also, the TMZ-driven mechanisms of cell death are independent of ferroptosis and recent studies indicate that TMZ induces an autophagy mechanism [27, 28]. A reason for the species differences for the multitox-approach may lay in differences in xCT levels. Indeed, human glioma cells show higher xCT expression levels compared to rodent gliomas (Supplementary Figure S2). This could make human gliomas more vulnerable towards xCT blocking strategies, since human gliomas may be more dependent on the xCT function. However, other pharmacological features might influence the susceptibility for the multitox-approach as well and it is conceivable that side-effects due to multiple targets could also account for these species-specific results.

The multitox-approach showed multiplicative effects on human gliomas. In this study we did not investigate the impact of this multitox strategy on the tumor microenvironment. This is an important parameter since in particular brain tumors are clinically dreaded for their microenvironmental disturbances. Thus, future studies will provide evidence whether xCT inhibition is a valid strategy in supporting clinically established chemotherapeutic agents.

## MATERIALS AND METHODS

### Chemicals

Temozolomide (TMZ) and Sulfasalazine (SAS) were purchased from Sigma-Aldrich (Taufkirchen, Germany). Erastin was purchased from Hycultec GmbH (Beutelsbach, Germany). Sorafenib was purchased from LC Laboratories (Woburn, USA). S-4-Carboxyphenylglycine was purchased from ACRIS Antibodies (Herford, Germany). Temozolomid was solved under sterile conditions in dimethylsulphoxide (DMSO) to concentration of 300 mM. Sulfasalazine was dissolved in 400 mM ammonium hydroxide under sterile conditions to concentration of 200 mM. Erastin and Sorafenib were dissolved in DMSO under sterile conditions to concentration of 100 mM. S-4-CPG was solved under sterile conditions in 1 M sodium hydroxide to concentration of 100 mM. Deferoxamine (DFO) and Ferrostatin-1 (Ferr-1) were purchased from Sigma-Aldrich (Taufkirchen, Germany). Deferoxamine was dissolved in water under sterile conditions to a concentration of 50 mM. Ferrostatin-1 was prepared in 50% DMSO/water under sterile conditions to a concentration of 50 mM.

### Cell culture, transfection

Glioma cell line F98 was obtained from ATCC/LGC-2397 (Germany). Primary rat astrocytes were prepared from up to one month old Wistar rats. All cells were cultured under standard humidified conditions (37°C, 5% CO_2_) with Dulbecco's Modified Eagle Medium (DMEM; Biochrom, Berlin, Germany) supplemented with 10% fetale bovine serum (Biochrom, Berlin, Germany), 1% Penicillin/Streptomycin (Biochrom, Berlin, Germany) and 1% Glutamax (Gibco/Invitrogen, California, USA). Cells were passaged at approx. 80% confluence. Cells were trypsinized after PBS wash step. After centrifugation (900 rpm for 5 min) cells were plated out in culture flask.

Cell lines were transfected according to Roti-Fect manufacturer's protocol (Carl Roth, Karlsruhe, Germany). Briefly, cells were plated at 10.0000 cells/well in 6-well plates and held under standard conditions. 18 h after seeding transfection was performed. Transfected cells were selected with geneticin sulfate 418 (Sigma, St.Louis, USA) and fluorescence-activated cell sorting.

### Expression and knock down vectors cloning

Human and rodent expression constructs were cloned as described previously in Savaskan et al., 2008 [8]). For sequence alignments and homology searches of xCT we utilized the www.ncbi.nlm.nih.gov database and A Plasmid editor software (ApE; MW Davis, Utah, USA). All orthologous sequences of xCT (human, mouse and rat) are deposited at the NCBI database (Human xCT GenBank accession no. AF252872; Rattus norvegicus xCT GenBank accession no. NM001107673; Mus musculus xCT GenBank Accession no. AB022345). For construct cloning we cloned fragments by PCR and inserted the resulting amplicons into the pEGFP (Takara, Heidelberg, Germany) vector. According to the critera of Ui-Tei et al., 2004 [29] three 19-mer short interfering RNAs were chosen for RNA interference with rat xCT transcripts (GenBank acc. NM001107673). Cloning of the synthetic oligonucleotids into the pSuperGFP vector (pS-GFP; OligoEngine) was performed by digesting the empty vector with EcoR1 and Xho1 according to the manufacturer's instruction. Cells were transfected at low density (<20.000 cells/cm^2^) and expression analysis was performed as Savaskan et al., 2008 [8] described.

### Cell viability analysis and toxicity assays

The cell viability assay was performed using 3(4,5 dimethylthiazol)-2,5 diphenyltetrazolium (MTT) assay according to Hatipoglu et al., 2015 [30] the cell viability was measured. 3.000 cells/well were plated in 96 well-plates one hours prior to the drug treatment. In case of the primary rat astrocytes culture, 4.000 cells/well were plated in 96 well-plates and the drug treatment were done after 4 days. On the fourth day after treatment cells were incubated with MTT solution (Roth, Karlsruhe, Germany) (5 mg/ml) for 4 h at 37°C, 5% CO_2_. For the ferroptosis measurement 20.000cells/96well were seeded, drug treatment occurred 1 h after seeding and the MTT solution was added after 24 h of incubation. The lysis of the cells occurred with 100 μl isopropanol + 0.1 N HCl. The optical density of each well was determined using the microplate reader Tecan Infinite F50 (Crailsheim, Germany) set to 550 nm (wavelength correction set to 690 nm) using Magellan software. Plates were normally read within 1 h of adding after lysis. Control was cells without drugs. The viability of the cells was expressed as the percentage of control. Assays were performed on at least three independent experiments.

### Cell death assay and apoptosis analysis

Cells were seeded 80000 cells/well in 6 well - plates five hours prior to the drug treatment. Cell death assay was performed on the fourth day. Cells were incubated with propidium iodide staining (PI) purchased from Molecular Probes (Invitrogen, Darmstadt, Germany). Cells were stained 20 min [1 μg/ml]. Apoptosis analysis was conducted with Wright staining. Therefore cells were seeded and treated the same way as the cell death assay. Cells were washed with PBS and then fixed and subjected to the Wright staining according to the manufacturer's instructions (Sigma-Aldrich, Taufkirchen, Germany). After staining, cells were dried and embedded. Afterwards morphological features of apoptotic cells were observed under an Olympus x71 and images were taken with cell-F software (Olympus, Tokyo, Japan). The same equipment was used for the cell death assay.

### Primary neurons-astrocytes isolation

Hippocampal neuronal cultures were prepared from one to four days old Wistar rats as described by Ghoochani et al., 2015. [31] Briefly, newborn rats were sacrificed by. Hippocampi were removed from the brain and transferred into ice cold Hank's salt solution, and the dentate gyrus was cut away. After digestion with trypsin (5 mg/ml) cells were triturated mechanically and plated in MEM medium, supplemented with 10% fetal calf serum and 2% B27 Supplement (all from Invitrogen, Taufkirchen). In brief, the culture medium was removed and replaced with Neurobasal A (Invitrogen, Taufkirchen). [32] Neurons were stained with beta-III-tubulin (1:500, Promega, Madison, Wisconsin, USA), astrocytes with GFAP (1:500, Dako, Glostrup, Denmark) and counterstained with Hoechst 33258 (1:10.000, Life Technologies, Darmstadt, Germany). Images were taken by an Axio Observer with the Zen Software (Zeiss, Oberkochen, Germany).

### Cell cycle analysis

80.000 cells/well were seeded in 6-well – plates and treated 1 h later with drugs. Cell cycle analysis was performed on the fourth day with Flow Cytometer BD FACSCanto II (BD Bioscience, Heidelberg, Germany) according to the protocol described by Fan et al., 2014. [33] Cells and media supernatant were collected. The pellet were washed with PBS and afterwards resuspended in PI-Hypotonic lysis buffer (PI-LB: 0,1% sodium citrate, 0,1% Triton X-100, 100 μg/ml RNAse). Cell cycle analyses were performed within 2 h after adding 7-AAD (7-aminoactinomycin D, Molecular Probes, Invitrogen, Darmstadt, Germany). Analyses were carried out with Flowing Software 2 (Turku Center for Biotechnology, University Turku, Finland).

### Amino acid profiling of glioma conditioned medium

Cells were seeded in 12 - well plates at a density of 200.000 cells/well in DMEM supplemented with 10% FBS, 1% P/S and 1% Glutamax. After incubation overnight the cells were 80% confluent. The medium was changed to DMEM without supplements and drugs were added. After incubating for another 12 h, medium was collected and measurement was performed by HPLC. Amino acids were analysed by ion - exchange chromatography and post - column ninhydrin derivatization technique using a fully automated amino acids analyzer (Biochrom 30+, Laborservice Onken, Gründau, Germany). For the amino acid analysis, 100 μL of sample was deproteinised with 100 μL of 10% sulphosalicylic acids. 20 μL of this supernatant was then loaded by the autosampler into a cation - exchange resin - filled column. Three independent experiments were performed.

### RNA isolation and qRT - PCR experiments

F98 xCT modulated cells as well as the F98 wildtype and U251 wildtype cells were cultured in T75 flasks and harvest at 80% confluency. Cells were washed with PBS and Trizol (Peqlab, Erlangen, Germany) was added. Cells were collected RNA was isolated according to the manufacture's protocol. RNA concentration was quantified by NanoVue™ Plus Spectrophotometer (GE Healthcare, UK). cDNA was synthesized with 1 μg of total RNA using DyNAmo cDNA Synthesis Kit (Biozym, Hessisch Oldendorf, Germany) according to the manufacturer's protocol. Real-time (DyNAmo ColorFlash SYBR Green qPCR Kit) PCR was performed in a LightCycler® 480 (Roche Applied Sciences) according to the manufacturer's protocol (Biozym, Hessisch Oldendorf, Germany). The oligos used in this study are: mouse/rat xCT forward primer: TGCTGGCTTTTGTTCGAGTCT; mouse/rat xCT reverse primer: GCAGTAGCTCCAGGGCGTA. Human xCT forward primer: CAGTAGCTGCAGGGCGTA; human xCT reverse primer: ACCCGCTGTTGTACGAGTC. GAPDH forward primer: TGCACCACCAACTGCTTAGC; GAPDH reverse primer: GGCATGGACTGTGGTCATGA. Real time cycling parameters: Initial activation step (95°C, 15 min), cycling step (denaturation 94°C, 15 s; annealing 60°C, 30 s; and extension 72°C, 30 s X 45 cycles), followed by a melting curve analysis to confirm specificity of the PCR. All samples were assessed in relation to the levels of GAPDH expression as an internal control. Q-PCR data were assessed and reported according to the ΔΔCt method. Data from at least five determinations (means ± SEM) are expressed as relative expression level.

### Statistical analysis

Analysis was performed using unpaired Student's *t-*test (MS Excel) as well as a two- and one-way anova (Graph Pad). The level of significance was set at *p < 0.05. Error bars represent ± SD as well as ± SEM.

## SUPPLEMENTARY FIGURES


