# Supplementary material for: Temozolomide toxicity operates in a xCT/SLC7a11 dependent manner and is fostered by ferroptosis
Source: Oncotarget. 2016 Sep 6;7(46):74630–47. doi: 10.18632/oncotarget.11858 (PMC5342691; doi:10.18632/oncotarget.11858)
Supplement: Supplementary file 1 [file oncotarget-07-74630-s001.pdf]

Temozolomide toxicity operates in a xCT/SLC7a11 dependent manner and is fostered by ferroptosis

SUPPLEMENTARY FIGURES

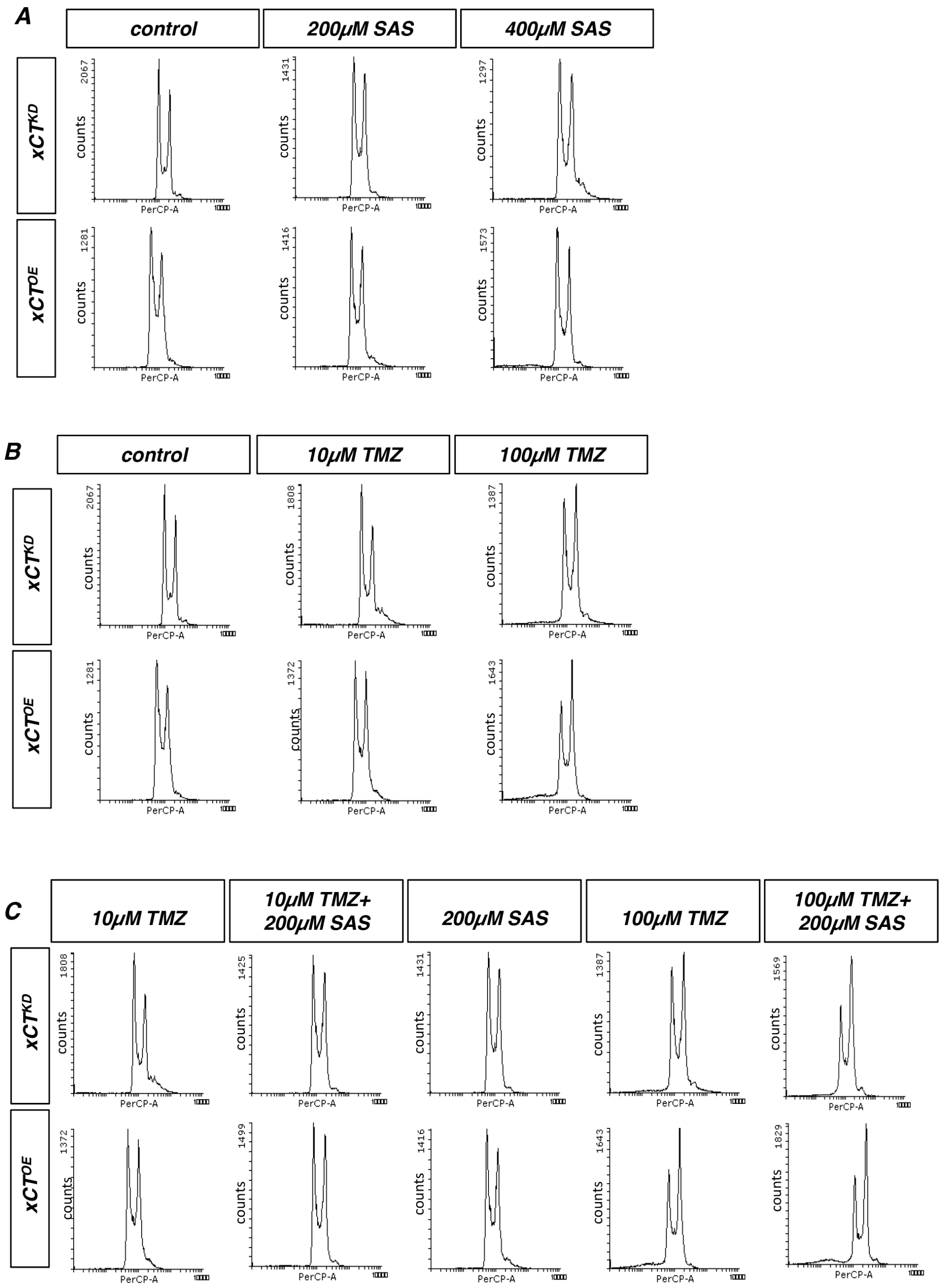

Supplementary Figure S1: Cell cycle and apoptosis analysis of glioma cells treated with temozolomide (TMZ) and sulfasalazine (SAS). A. Cell cycle analysis of glioma cells (F98) overexpressing xCT (xCT^{OE}) or silencing xCT (xCT^{KD}) were examined after SAS treatment. Apoptosis increased in a concentration dependent manner. **B, C.** Cell cycle of RNAi mediated xCT knock down cells (xCT^{KD}) and overexpressing xCT (xCT^{OE}) cells were measured after the treatment with ± SAS and ± TMZ and their combination. Differences were considered statistically significant with values given as mean ± SD ($n \geq 3$ per group; unpaired two-sided *t*-test, $p < 0.05$).

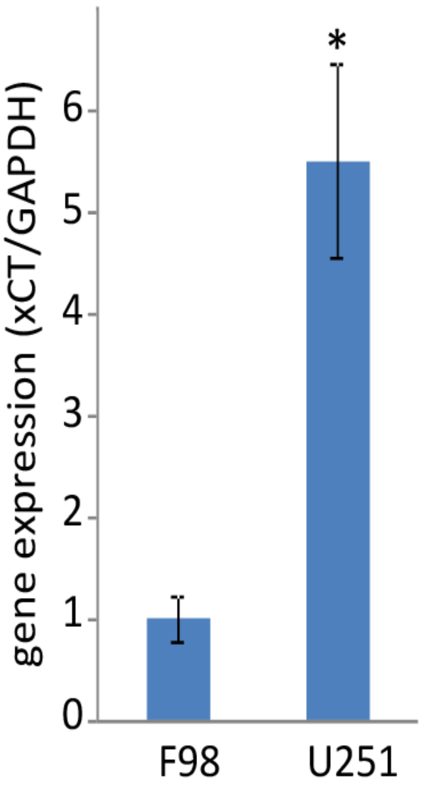

Supplementary Figure S2: xCT expression profiles in human and rat gliomas. A. Analysis of the xCT levels of wildtype rat F98 cells and wildtype human U251 glioma cells. Wildtype U251 cells express more xCT in comparison to F98 cells. Differences were considered statistically significant with values given as mean ± SEM ($n \geq 10$ per group; unpaired two-sided *t*-test, $p < 0.05$).
